# Cerebral microvascular density, blood-brain barrier permeability, and support for neuroinflammation indicate early aging in a Marfan syndrome mouse model

**DOI:** 10.3389/fphys.2024.1457034

**Published:** 2025-01-31

**Authors:** Tala Curry-Koski, Liam P. Curtin, Mitra Esfandiarei, Theresa Currier Thomas

**Affiliations:** ^1^ Phoenix Children’s Research Institute, Department of Child Health, College of Medicine-Phoenix, University of Arizona, Phoenix, AZ, United States; ^2^ Translational Neurotrauma and Neurochemistry Laboratory, Barrow Neurological Institute at Phoenix Children’s Hospital, Phoenix, AZ, United States; ^3^ College of Graduate Studies, Midwestern University, Glendale, AZ, United States; ^4^ Faculty of Medicine, University of British Columbia, Vancouver, BC, Canada; ^5^ Phoenix VA Healthcare System, Phoenix, AZ, United States

**Keywords:** Marfan syndrome, microvascular density, blood-brain barrier, neuroinflammation, neuropathology, premature aging

## Abstract

**Introduction:**

Marfan Syndrome (MFS) is a connective tissue disorder due to mutations in fibrillin-1 (*Fbn1*), where a *Fbn1* missense mutation (*Fbn1* ^*C1039G/+*^) can result in systemic increases in the bioavailability and signaling of transforming growth factor-β (TGF-β). In a well-established mouse model of MFS (*Fbn1* ^*C1041G/+*^), pre-mature aging of the aortic wall and the progression of aortic root aneurysm are observed by 6-month-of-age. TGF-β signaling has been implicated in cerebrovascular dysfunction, loss of blood-brain barrier (BBB) integrity, and age-related neuroinflammation. We have reported that pre-mature vascular aging in MFS mice could extend to cerebrovasculature, where peak blood flow velocity in the posterior cerebral artery (PCA) of 6-month-old (6M) MFS mice was reduced, similarly to 12-month-old (12M) control mice. Case studies of MFS patients have documented neurovascular manifestations, including intracranial aneurysms, stroke, arterial tortuosity, as well as headaches and migraines, with reported incidences of pain and chronic fatigue. Despite these significant clinical observations, investigation into cerebrovascular dysfunction and neuropathology in MFS remains limited.

**Methods:**

Using 6M-control (*C57BL/6*) and 6M-MFS (*Fbn1* ^*C1041G/+*^) and healthy 12M-control male and female mice, we test the hypothesis that abnormal Fbn1 protein expression is associated with altered cerebral microvascular density, BBB permeability, and neuroinflammation in the PCA-perfused hippocampus, all indicative of a pre-mature aging brain phenotype. Glut1 immunostaining was used to quantify microvascular density, IgG staining to assess BBB permeability, and microglial counts to evaluate neuroinflammation.

**Results:**

Using Glut1 staining, 6M-MFS mice and 12M-CTRL similarly present decreased microvascular density in the dentate gyrus (DG), cornu ammonis 1 (CA1), and cornu ammonis 3 (CA3) regions of the hippocampus. 6M-MFS mice exhibit increased BBB permeability in the DG and CA3 as evident by Immunoglobulin G (IgG) staining. No differences were detected between 6M and 12M-CTRL mice. 6M-MFS mice show a higher number of microglia in the hippocampus compared to age-matched control mice, a pattern resembling that of 12M-CTRL mice.

**Discussion:**

This study represents the first known investigation into neuropathology in a mouse model of MFS and indicates that the pathophysiology underlying MFS leads to a systemic pre-mature aging phenotype. This study is crucial for identifying and understanding MFS-associated neurovascular and neurological abnormalities, underscoring the need for research aimed at improving the quality of life and managing pre-mature aging symptoms in MFS and related connective tissue disorders.

## Introduction

Marfan Syndrome (MFS) is the most common monogenetic autosomal-dominant disorder of connective tissue due to mutations in the gene encoding for fibrillin-1 (*Fbn1*) that presents with manifestations in many organs and systems in the body, resembling phenotypes of premature aging (Canadas et al., 2010a; Canadas et al., 2010b). The *Fbn1* gene encodes for a large glycoprotein, a structural component of microfibrils in the extracellular matrix (ECM) (Sengle and Sakai, 2015). Therefore, *Fbn1* mutations can result in a wide range of complications, particularly those associated with connective tissues that require microfibril scaffolding, such as elastin. Thus, the cardiovascular, musculoskeletal, and pulmonary systems are commonly affected, with ascending aortic aneurysm and rupture being the most life-threatening manifestation. MFS vascular complications are attributed to compromised ECM, medial elastin fiber fragmentation, and endothelial dysfunction, all leading to vascular wall stiffening and weakening, contributing to the vascular aging phenotype (Jimenez-Altayo et al., 2017; Cui et al., 2014; Gibson et al., 1985; Chung et al., 2007a; Chung et al., 2007b; Syyong et al., 2009).

Fbn1 protein acts by binding and sequestering the latent form of the cytokine transforming growth factor-β (TGF-β) via a latent binding protein (LTBP), forming a large sequestering domain. However, the abnormal and dysfunctional Fbn1 protein fails to sequester TGF-β in its inactive form. This uncontrolled release of bioavailable TGF-β facilitates the activation of downstream signaling molecules involved in inflammatory responses, including matrix metalloproteinases (MMPs), which contribute to the degradation of elastin, a key and crucial component of the ECM structure within the wall of elastic arteries (Chung et al., 2007a; Chung et al., 2007b; Benke et al., 2013). Indeed, previous studies in MFS patients and experimental mouse models have confirmed increased expression and activity of MMP-2/-9 in the peripherary (Cui et al., 2014; Chung et al., 2007a; Chung et al., 2007b; Sellers et al., 2018).

Cerebrovascular aging increases the risk for neurological deficits such as cognitive decline, dementia, stroke, and cerebral aneurysms (Vasilevko et al., 2010; Abrahamson and Ikonomovic, 2020; Wilson and Matschinsky, 2020). MFS patients and mice display accelerated and pre-mature aging phenotypes within the peripheral vasculature, including increased vascular wall stiffness, endothelial dysfunction, decreased smooth muscle contractility, mitochondrial dysfunction, increased inflammatory infiltrates, and elevated reactive oxygen species (ROS) production within the vessel wall (Chung et al., 2007a; Chung et al., 2007b; Syyong et al., 2009; Gibson et al., 2017; Jiménez-Altayó et al., 2018; Verhagen et al., 2021). This pre-mature aging phenotype has been observed and supported in multiple systems of the body, including the integumentary and circulatory systems (Curry et al., 2024; Tehrani et al., 2020). MFS patients experience other age-related changes such as worsening joint pain and stiffness, vision problems, hearing loss, skin elasticity, and lung complications (Canadas et al., 2010a; Canadas et al., 2010b; Tehrani et al., 2020; Andonian et al., 2021; Handisides et al., 2019). MFS-associated symptomology are also implicated in pathophysiological manifestations related to aging, such as inflammation, ROS production, arterial remodeling, and elastolysis all seen in the periphery thus far. Publications from our lab further supported this pre-mature aging phenotype in cerebral vascular function in transgenic mice with a *Fbn1* mutation *(MFS mice)*. Specifically, we demonstrated that the peak blood flow velocity in the posterior cerebral artery (PCA) of 6-month-old male MFS mice is reduced, showing similarities to that of middle-aged, 12-month-old control male mice (Curry et al., 2024).

Cerebral blood flow is a critical physiological process required to ensure the brain receives the necessary oxygen and nutrients to function properly. Microvascular density, the number of microvessels in a given area such as the brain, plays a key role in cerebral blood flow. For instance, microvascular density is integral to regulating cerebral blood flow by influencing capacity and efficiency of blood delivery to the brain (Li et al., 2023). Factors influencing microvascular density include but are not limited to angiogenic factors, hypoxia, and vascular stress. Microvascular density in the brain is commonly assessed using histological methods, including staining of glucose transporter 1 (Glut1), a protein predominately expressed on endothelial cells of brain capillaries that facilitates glucose transport across the blood-brain barrier (BBB) (Harik, 1992; DH and AW, 1999). Aging is associated with a reduction in microvascular density, contributing to decreased cerebral blood flow, which can lead to impaired neurological function (Li et al., 2023; Vorbrodt et al., 1999; Vorbrodt et al., 2001). The MFS-associated premature aging phenotype and decreased cerebral blood flow velocity warrants further investigation to evaluate microvascular density in the brain.

The BBB is the first line of defense and a key player in maintaining homeostasis in the central nervous system. This highly selective and semipermeable barrier is made up of different cell types and proteins comprising endothelial cells that line the lumen of the blood vessel and are interconnected by a series of tight junctions to ensure strict segregation of the cerebral and vascular environments. Breakdown of the tight junction components, claudins and occludins could lead to an increase in permeability. The basal lamina of the endothelial cells adds another degree of separation between the two environments. Pericytes also line the endothelial cells involved in regulating blood flow and maintaining the BBB (Attwell et al., 2016). In addition, astrocytic end feet wrap around the vessel and further regulate the passage of different molecules, regulating the cerebral environment. Increased BBB permeability is a phenomenon associated with aging, leading to various theories about the pathogenesis of aging-related neuropathologies such as dementia, Parkinson’s disease, and mild cognitive impairment, suggesting a link between the compromised integrity of the BBB and the development of these neurological conditions (Cheng et al., 2024; Nelson et al., 2024; Candelario-Jalil et al., 2022). Vascular dysfunction has also been associated with increased risk for BBB permeability (Ricci et al., 2024; Hansra et al., 2024). In addition, increased MMPs −2/-9 expression has been linked to disruption of tight junctions and increased BBB permeability (Hansra et al., 2024; Ji et al., 2023; Rempe et al., 2016). It is important to note that MMP-2/-9 are found to be upregulated in the blood and aortic tissue of MFS patients and mouse models. This makes it plausible that their expression might also be elevated in the cerebrovasculature and brain of MFS mice, potentially contributing to the observed neuropathological changes (Rempe et al., 2016; Segura et al., 1998; Kunz, 2007; Galis and Khatri, 2002).

Microglia are the resident immune cells of the brain and are significantly involved in neuroinflammatory responses, phagocytosis, homeostasis, and regulation of synapses. Neuroinflammation is characterized by the activation of microglia, which is marked by changes in morphology and an increased number of Ionized Calcium-Binding Adapter Molecule one (iba-1)-positive cell bodies. However, excessive and chronic activation of microglia, as observed in aging and neurodegenerative diseases, can result in impaired microglial function and neuronal damage, leading to neurodegeneration and cognitive decline (Angelova and Brown, 2019; Triviño and von Bernhardi, 2021). Increased permeability of the BBB is a well-recognized factor contributing to neuroinflammation, creating a vicious cycle where neuroinflammation further increases BBB permeability (Parsi et al., 2024). In addition, increased MMPs −2/-9 expression has been shown to exacerbate BBB permeability and stimulate neuroinflammation, further augmenting the cycle of damage and inflammation within the brain (Chopra et al., 2015).

While aortopathy in MFS patients and animal models has been extensively researched by numerous groups, our understanding of other manifestations of MFS remains significantly limited. Currently, there is a notable gap in preclinical investigation into these manifestations and their possible impact on neurological outcomes. This lack of research highlights the need for a broader exploration of MFS beyond its cardiovascular implications, to fully understand the syndrome’s comprehensive effects on health and quality of life. In the present study, we aimed to test the hypothesis that a missense mutation in the *Fbn1* gene (*Fbn1* ^*C1041G/+*^) results in changes in neuropathology in the well establish MFS mouse model. Furthermore, we aimed to determine whether the outcomes observed in 6-month-old MFS (6M-MFS) mice more closely resemble the phenotypes seen in 12-month-old healthy control mice (12M-CTRL), highlighting the potential premature and early aging brain phenotype in the MFS mouse model.

## Methods

### Experimental animals

Animal care was conducted according to the National Research Council Guide for the Care and Use of Laboratory Animals and the Guidelines for Animal Experiments and the Midwestern University Animal Care and Use Committee [IACUC protocols AZ-3006 & AZ-2936]. Mice were group-housed (up to five mice per cage) in a 12/12 h light-dark cycle with food and water available *ad libitum*. Breeding pairs were obtained from Jackson Laboratory and backcrossed for eight generations. A breeding colony of MFS mice was established and maintained at Midwestern University Animal Facility (IACUC breeding protocol AZ-2989). Mice used in this study were heterozygous for an *Fbn1* allele encoding a cysteine substitution in the epidermal growth factor-like domain in *Fbn1* to glycine (*Fbn1* ^*C1041G/+*^), giving the mice the most common type of mutation seen in MFS patients presented with vascular dysfunction and aortic root aneurysm. This study utilized adult male and female *Fbn1* ^*C1041G/+*^ (MFS) mice at 6 months of age, as well as male and female *C57BL/6* (CTRL) mice at 6 and 12 months of age. MFS mice present established and evident vascular defects (e.g., aortic wall elastin fragmentation, aortic root aneurysm, and increased aortic wall stiffness) by 6 months of age. This time point is equivalent to 30–35 human years and was compared to middle-aged (12-month-old) CTRL mice, to evaluate the MFS-associated accelerated vascular aging phenotype. These MFS mice are bred on a *C57BL/6* background, making *C57BL/6* mice the appropriate controls for this study. After genotyping via tail snip and PCR testing, an N = 4-5/group with combined male and female mice were randomly assigned to naïve 6M-old CTRL and MFS, and 12M-old CTRL mice for immunohistochemistry and utilized for all measures described. As the first known study to evaluate neuropathology in MFS mice, we included both male and female mice in order to evaluate these manifestations overall in MFS. Future investigations would benefit from including a larger sample size for both males and females in order to evaluate sex as a biological factor contributing to neuropathology. Pre-determined welfare exclusion criteria included removing any mouse from the study with visible wounds requiring veterinarian intervention or vocalization of pain that cannot be managed. No animals required exclusion due to welfare concerns.

### Immunohistochemistry

Mice were euthanized using 5% isoflurane inhalation followed by cervical dislocation, and brain tissue removed and hemisected (N = 4-5/group). One hemisphere of each brain was post-fixed in 4% paraformaldehyde, preserved in a sodium azide solution, and sent to Neuroscience Associates (Knoxville, TN) for proprietary embedding techniques, sectioning, and staining for Glut1, Iba1, and IgG. Coronal sections (35 µm thick) were collected sequentially into containers contained Antigen Preserve solution (50% PBS, pH 7.0, 50% ethylene glycol, 1% polyvinyl pyrrolidone). Glut1, a blood-brain barrier marker, was detected using a rabbit anti-Glut1 primary antibody (Millipore, Catalog# 07-1401; dilution 1:100,000) and a biotinylated goat anti-rabbit IgG secondary antibody (Vector, Catalog# BA-1000; dilution 1:1,000), then visualized using NiDAB as the chromogen. Iba1, a microglia and macrophage marker used to evaluate morphology as an indicator of neuroinflammatory phenotypes, was detected with a rabbit anti-Iba1 primary antibody (Abcam, Catalog# ab178846; dilution 1:75,000) and the same biotinylated goat anti-rabbit IgG secondary antibody (Vector, Catalog# BA-1000; dilution 1:1,000) then visualized using NiDAB. For IgG, a marker of BBB permeability, a biotinylated horse anti-mouse IgG primary antibody (Vector, Catalog# BA-2001; dilution 1:238,000) was used then visualized using DAB.

### Imaging and analysis

**FIGURE 1:**
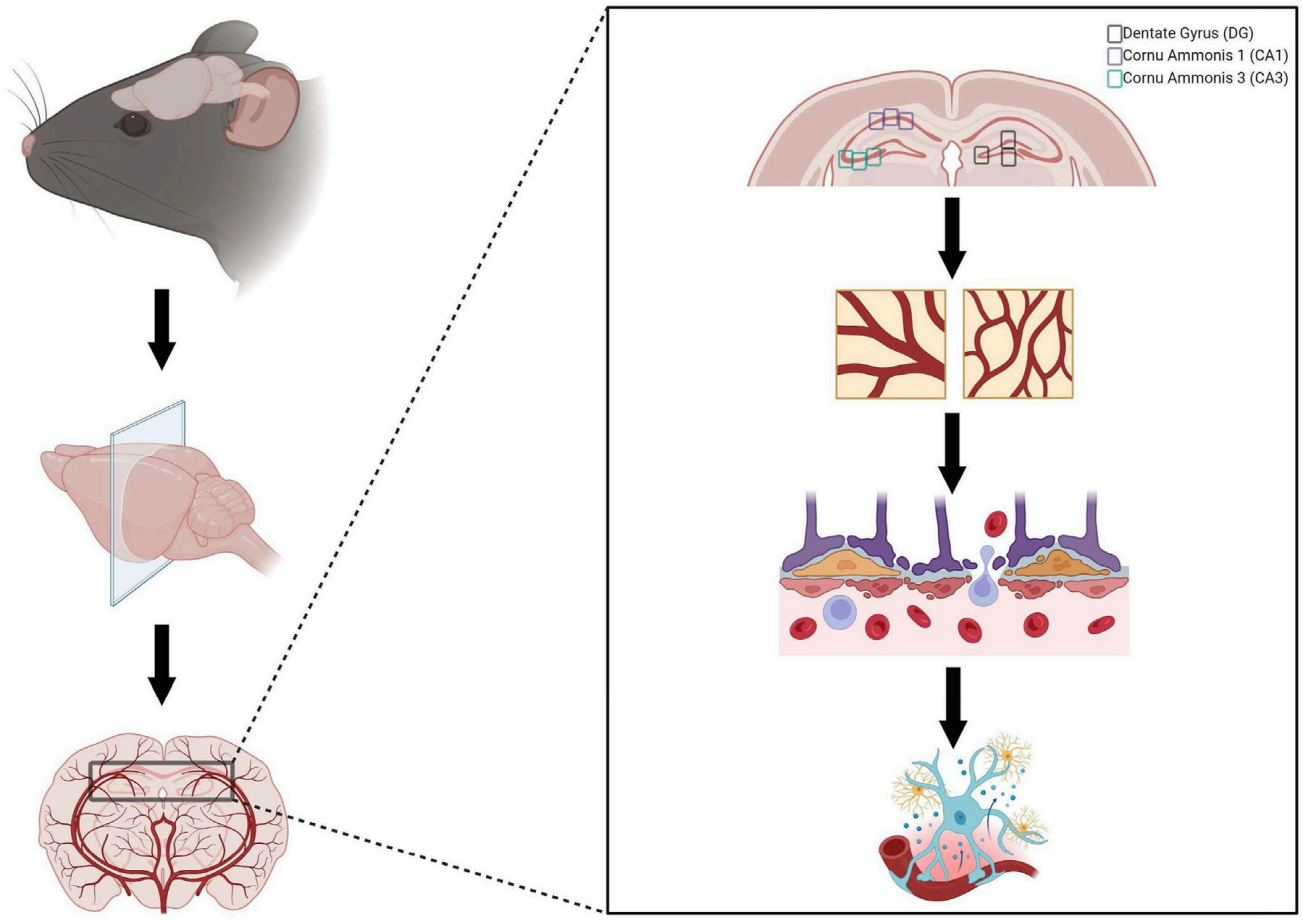
Representation of neuropathology evaluation. Brains were collected, post-fixed in 4% paraformaldehyde, blocked, sectioned, and stained by Neuroscience Associates (Knoxville, TN). Neuropathology was evaluated by investigating Glut1 staining to evaluate microvascular density, Immunoglobulin G (IgG) staining to assess blood-brain barrier permeability, and iba-1 staining to investigate neuroinflammation in three regions of interest within the hippocampus (dentate gyrus, cornu ammonis 1, and cornu ammonis 3). Created with BioRender.com.

The hippocampus, perfused by the PCA, was targeted and analysis was done in the DG, CA1, and CA3 of the hippocampus. For each stain, three images were taken per DG, CA1, and CA3, respectively, per coronal brain section, where three sections were assessed per animal, for a total of nine images per animal averaged for each data point in each region of interest (27 images per animal) (Figure 1). Altogether, due to the cohesive and simultaneous sectioning and staining technques utilied by NSA, these protocols minimized variability of staining or sectioning across samples.

Brightfield 40x images of the hippocampus were independently analyzed using ImageJ to assess the pixel density of Glut1 and IgG-stained sections. For Glut1 staining, percent area of staining was quantified by applying a thresholding technique that isolated darker staining areas, ensuring minor variations in intensity did not affect the evaluation. Regions of interest in the hippocampus were converted to 8-bit images, background-subtracted to achieve a light background, and inverted. Thresholding was then applied to render darker staining areas as black and lighter staining areas as white. The percent area of black pixels, representing stained regions, was measured and reported.

IgG staining was assessed similarly, with particle size adjusted to exclude particles greater than 5,000 pixels to remove vessels that may retain IgG. Circularity was set to 0.2–0.7 to focus on irregular shapes and exclude round particles that could result from the counterstain. This protocol was performed consistently across all experimental groups.

As previously described by our laboratory, the ImageJ Skeleton Analysis plugin was utilized to assess microglial branch points, endpoints, branch length, and the number of microglia soma as a global indication of neuroinflammation for Iba-1-stained sections (Beitchman et al., 2020; Bromberg et al., 2020). Iba1 staining was assessed by converting the images to 8-bit. Images were then adjusted for brightness/contrast, where the thresholding histogram was enclosed within the boundaries. An unsharp mask filter with a radius of three was run to better define the edges of microglial projections, and then converted to binary. The remove outliers function was set to a radius of one to remove speckling noise in the background. Images were then processed using the skeletonize function and then the Skeleton analysis plugin utilized.

### Statistical analysis

All graphs and statistical significance were created and determined with GraphPad Prism software. The sample size for experimental groups was calculated based on desired endpoints where power was set at 90% and α = 0.05 utilizing the Mean ± SD from our and other previously published data (N = 4-5/group) (Beitchman et al., 2020). The study was powered to address two independent research questions: (1) the effect of MFS compared to Control at 6M and (2) the effect of aging on phenotype. As such, two distinct statistical approaches were initially employed to analyze the data. To streamline the presentation of data and address both research questions comprehensively, a one-way analysis of variance (ANOVA) test was performed on data sets with one independent variable (e.g., phenotype) comparing 6M-CTRL, 6M-MFS, and 12M-CTRL mice. This did not change the results or interpretation of the data. Tukey *post hoc* was performed on ANOVA tests. All data sets were tested for outliers utilizing ROUT (Q = 1%) and, if determined, were excluded. Furthermore, data sets were evaluated for Gaussian distribution to test for normality of residuals using the Kolmogorov-Smirnov test. Sex differences were not evaluated due to low sample sizes. Thus, we could not make reasonable statistical interpretations on sex as a biological factor in this study. Significance was determined as a *p*-value ≤ 0.05. All *p*-values are reported in the graphs, and raw data can be accessed by contacting the corresponding author. Personnel were blinded to genotype, age, and sex during analysis. Secondary analysis was performed by blinded personnel to replicate and confirm findings.

## Results

### Hippocampal Glut1 staining is decreased by age and genotype

**FIGURE 2:**
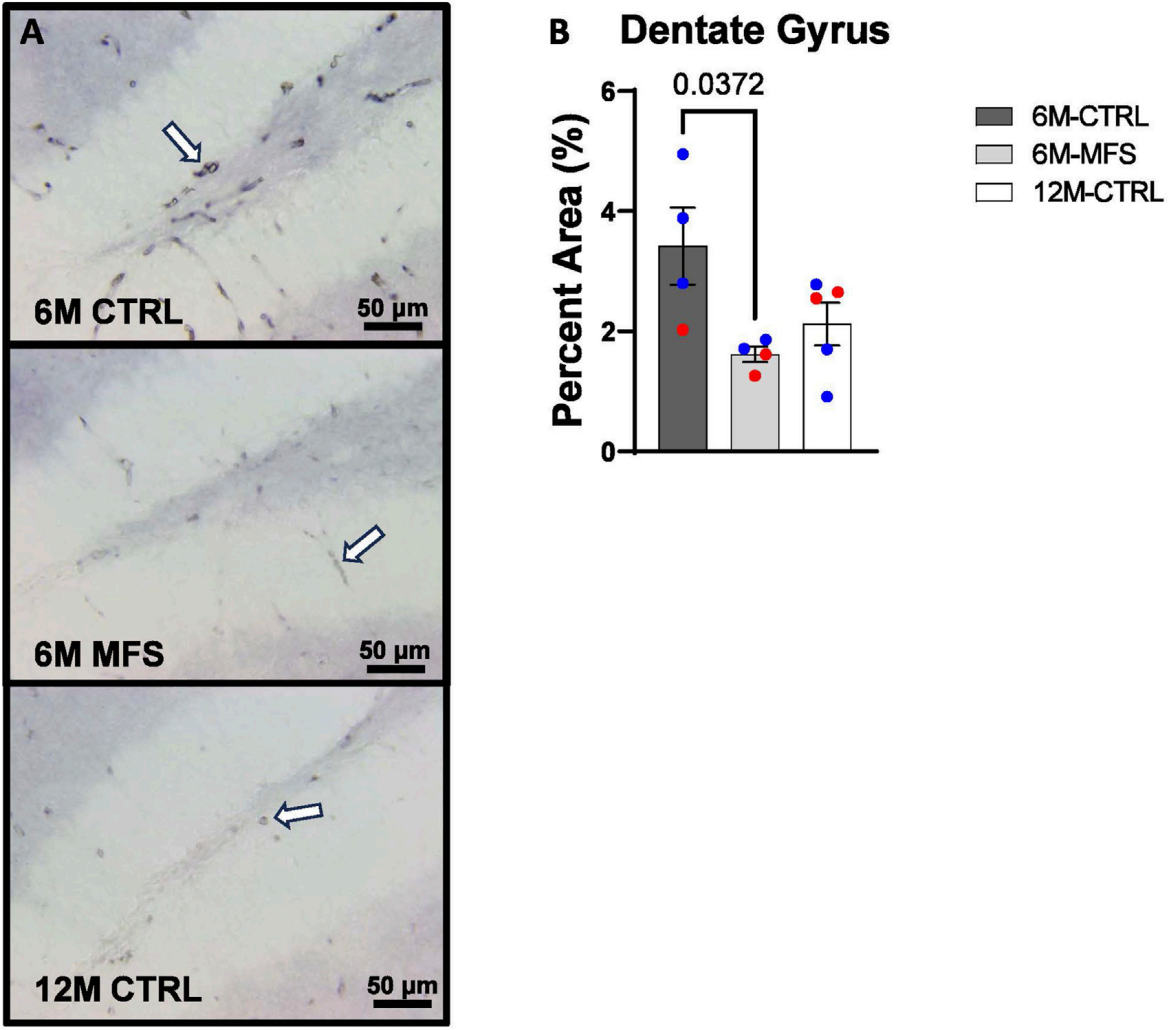
Glut1 staining in the Dentate Gyrus (DG) is decreased as a function of genotype and age, indicative of decreased microvascular density. **(A)** Representative images of Glut1 staining in the DG of the hippocampus in 6M-CTRL, 6M-MFS, and 12M-CTRL respectively. Examples of positive signals are represented by white arrows. **(B)** Glut1 staining is decreased in 6M-MFS DG compared to 6M- CTRL, indicative of decreased microvascular density. 6M-MFS compared to 12M-CTRL mice demonstrate no significant differences in Glut1 staining. Male mice are represented with blue data points and females are represented with red data points.

**FIGURE 3:**
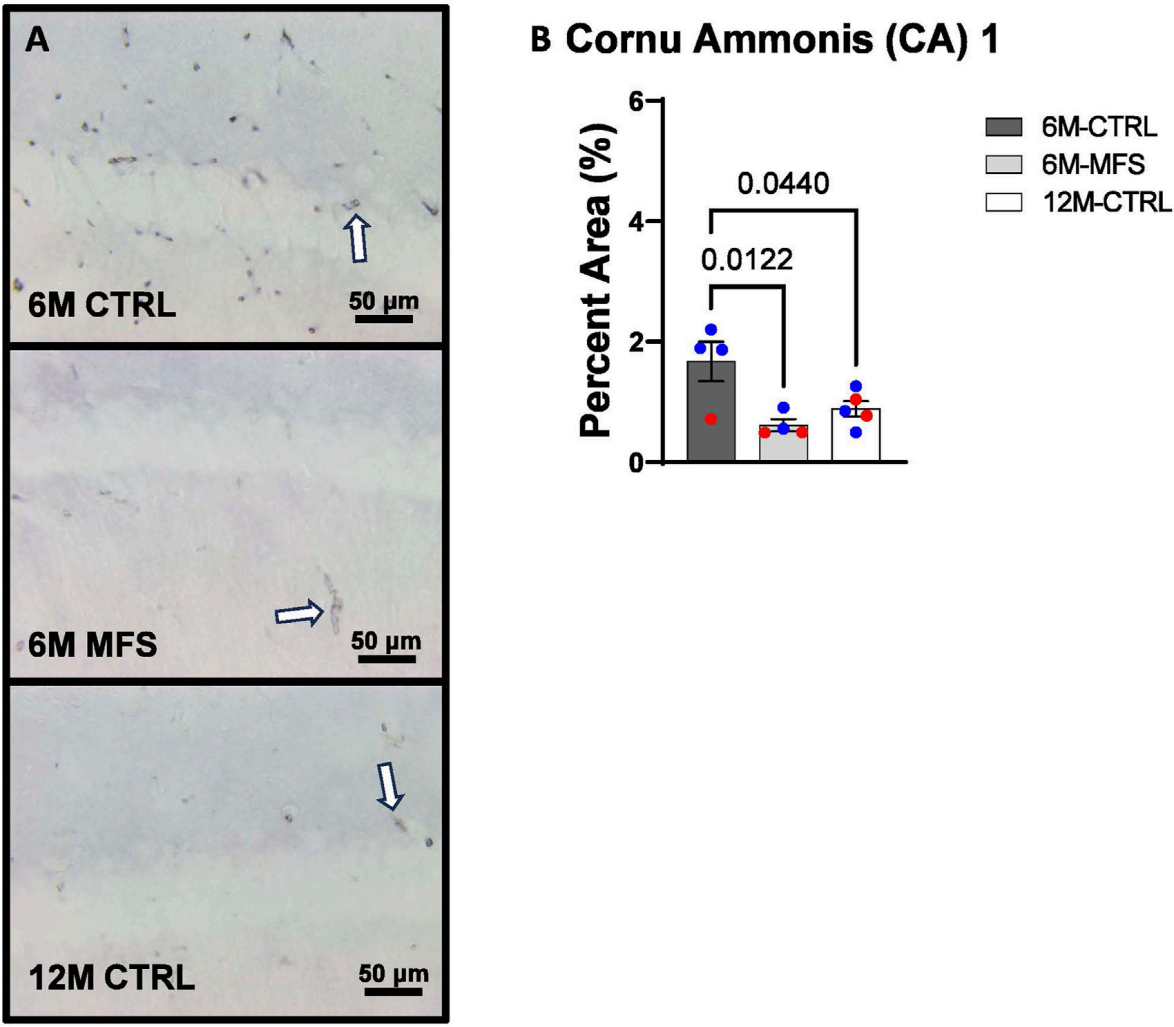
Glut1 staining in the cornu ammonis 1 (CA1) is decreased as a function of genotype and age, indicative of decreased microvascular density. **(A)** Representative images of Glut1 staining in the CA1 of the hippocampus in 6M-CTRL, 6M-MFS, and 12M-CTRL respectively. Examples of positive signals are represented by white arrows. **(B)** Glut1 staining is decreased in 6M-MFS CA1 compared to 6M-CTRL. 12M-CTRL CA1 demonstrate decreased Glut1 staining compared to 6M-CTRL, with no differences compared to 6M-MFS mice. Male mice are represented with blue data points and females are represented with red data points.

**FIGURE 4:**
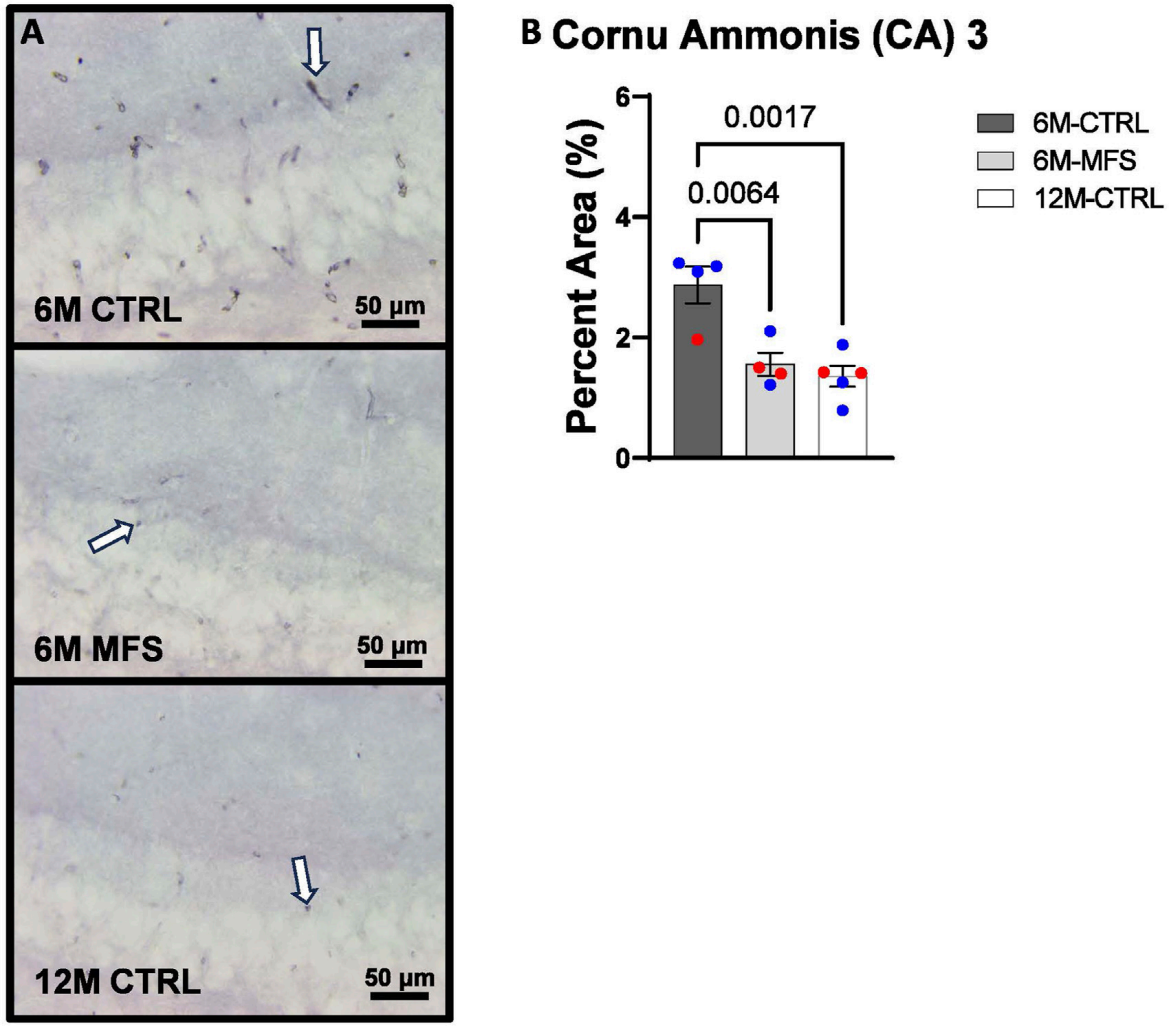
Glut1 staining in the cornu ammonis 3 (CA3) is decreased as a function of genotype and age, indicative of decreased microvascular density. **(A)** Representative images of Glut1 staining in the CA3 of the hippocampus in 6M-CTRL, 6M-MFS, and 12M-CTRL respectively. Examples of positive signals are represented by white arrows. **(B)** Glut1 staining is decreased in 6M-MFS CA3 compared to 6M-CTRL. 12M-CTRL CA3 demonstrate decreased Glut1 staining compared to 6M-CTRL, with no differences compared to 6M-MFS mice. Male mice are represented with blue data points and females are represented with red data points.

To investigate neurovascular alterations, we sought a method to evaluate gross morphological changes in hippocampal microvasculature. Glut1 staining was utilized to mark vascular endothelial cells, a well-known method for evaluating microvascular density (Figures 2A, 3A, 4A). 6M-MFS mice demonstrated decreased Glut1 staining in the dentate gyrus (DG) of the hippocampus compared to age-matched control mice (Figure 2B), yet no difference is seen when compared to 12M-CTRL mice (Figure 2B). Glut1 staining was decreased in 6M-MFS compared to 6M-CTRL (Figure 3B), and 12M-CTRL mice compared to 6M-CTRL mice in the cornu ammonis 1 (CA1) (Figure 3B). Glut1 staining was decreased in 6M-MFS compared to 6M-CTRL (Figure 4B), and 12M-CTRL mice compared to 6M-CTRL mice in the cornu ammonis 3 (CA3) (Figure 4B), while no differences were seen between 6M-MFS and 12M-CTRL mice.

### Blood-brain barrier permeability is increased as a function of genotype in the hippocampus

**FIGURE 5:**
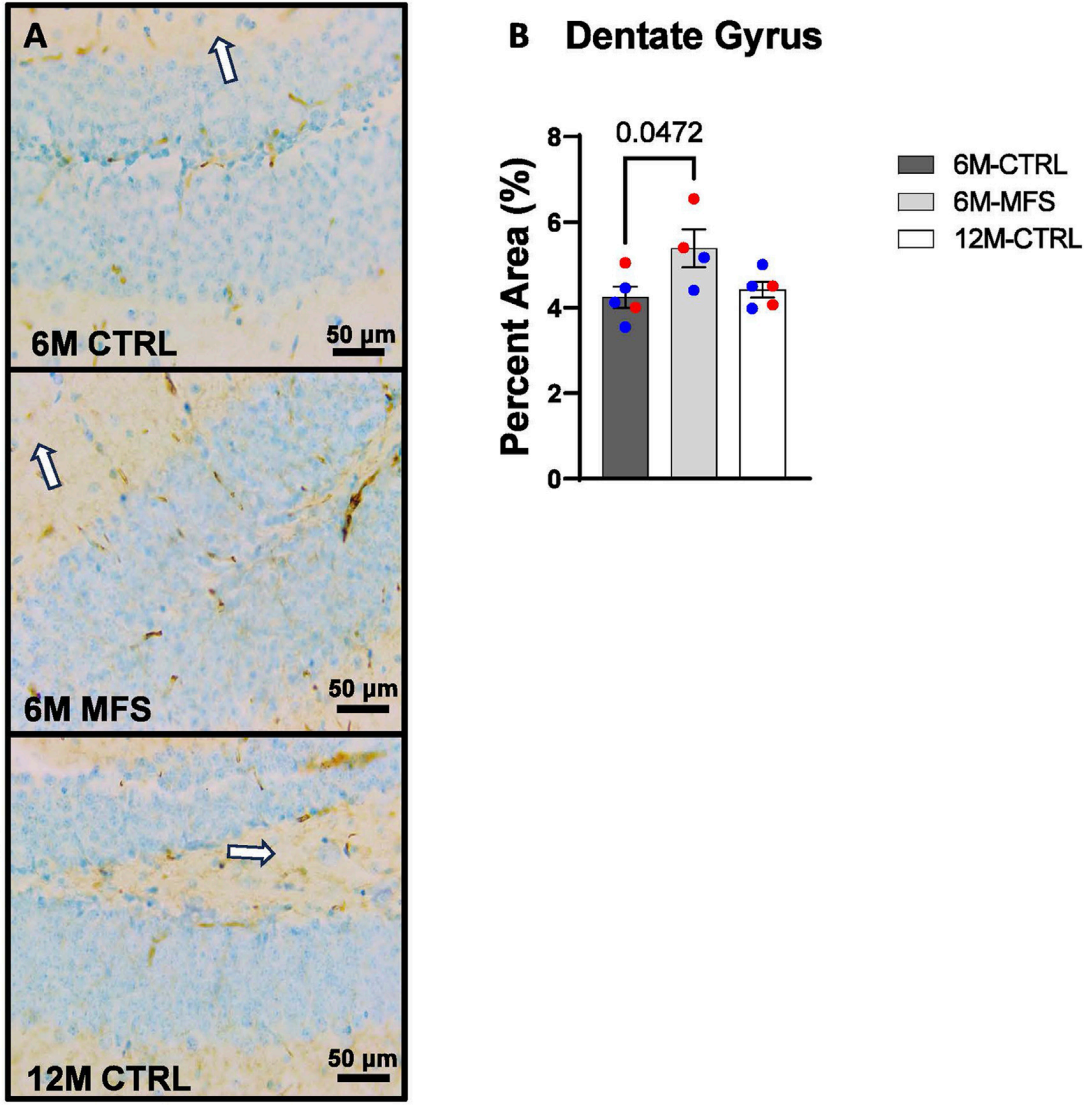
BBB permeability in the dentate gyrus (DG) of the hippocampus is increased as a function of genotype and age. **(A)** Representative images of IgG staining in the DG of the hippocampus in 6M- CTRL, 6M-MFS, and 12M-CTRL respectively. Examples of positive signals are represented by white arrows. **(B)** IgG staining is increased as a function of genotype, where 6M-MFS is increased compared to 6M-CTRL in the DG. No differences were seen between 6M-MFS and 12M-CTRL. Male mice are represented with blue data points and females are represented with red data points.

**FIGURE 6:**
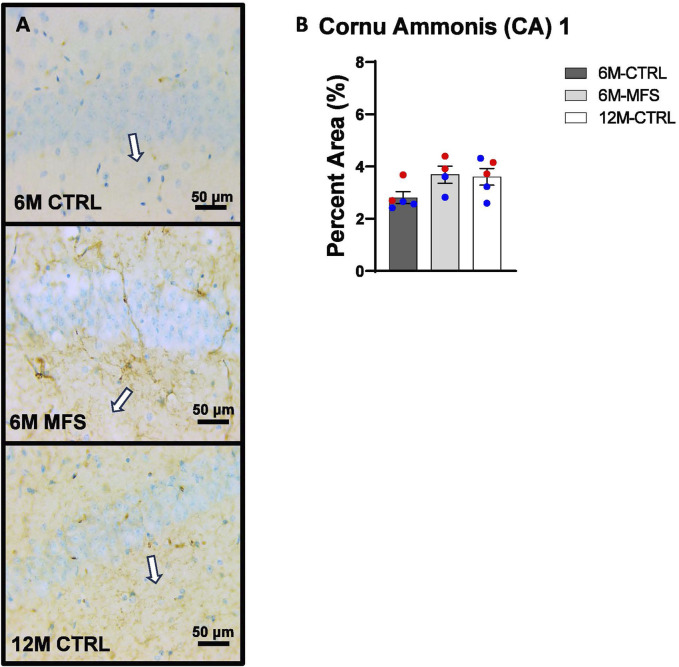
BBB permeability in the cornu ammonis 1 (CA1) of the hippocampus is increased as a function of genotype and age. **(A)** Representative images of IgG staining in the CA1 of the hippocampus in 6M-CTRL, 6M-MFS, and 12M-CTRL respectively. Examples of positive signals are represented by white arrows. **(B)** IgG staining is not significantly different in 6M-MFS and 12M-CTRL compared to 6M-CTRL mice in the CA1. Male mice are represented with blue data points and females are represented with red data points.

**FIGURE 7:**
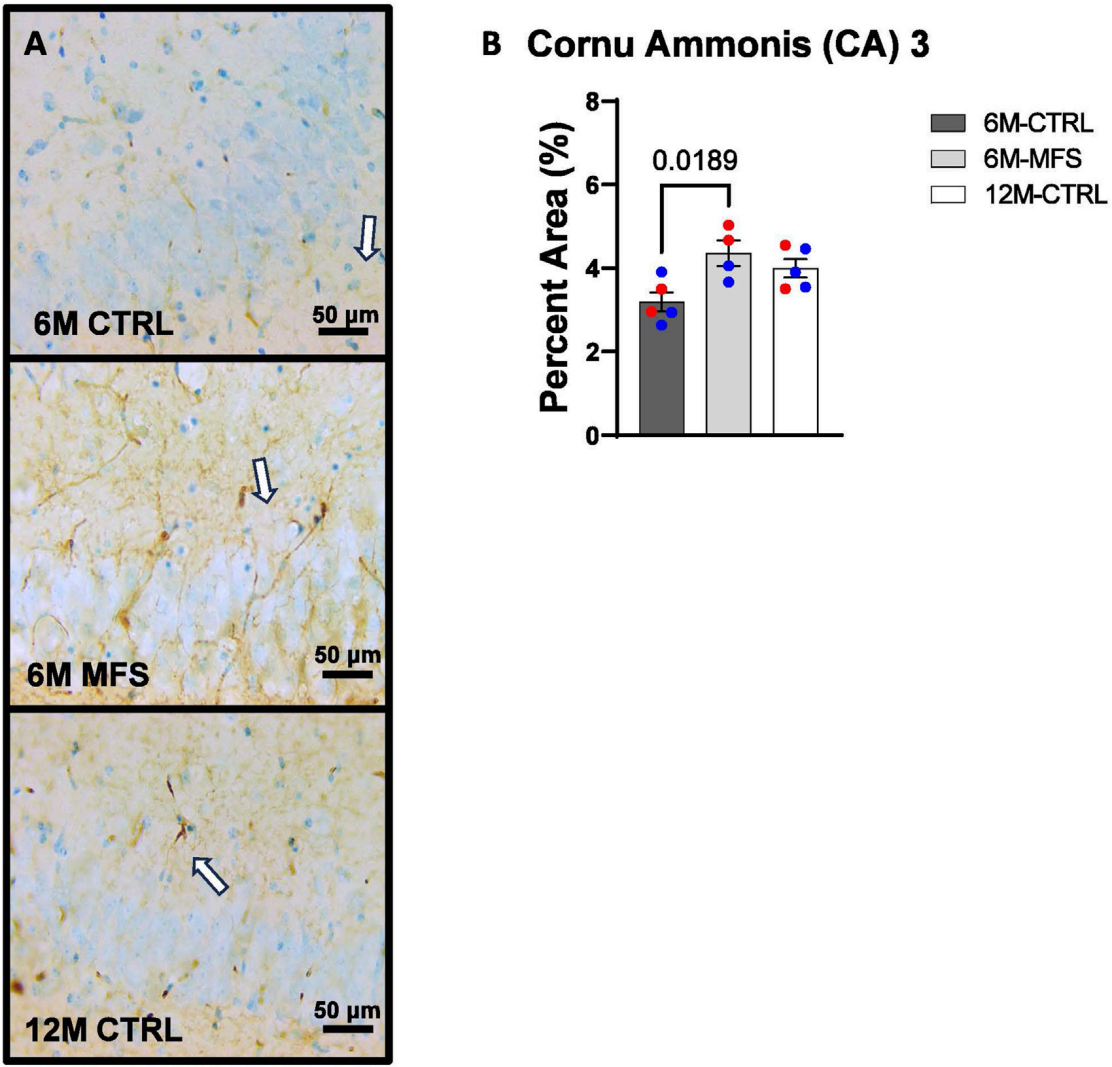
BBB permeability in the cornu ammonis 3 (CA3) of the hippocampus is increased as a function of genotype and age. **(A)** Representative images of IgG staining in the CA3 of the hippocampus in 6M-CTRL, 6M-MFS, and 12M-CTRL respectively. Examples of positive signals are represented by white arrows. **(B)** IgG staining is increased as a function of genotype, where 6M-MFS is increased compared to 6M-CTRL in the CA3. No differences were seen between 6M-MFS and 12M- CTRL. Male mice are represented with blue data points and females are represented with red data points.

In addition, we sought to evaluate BBB permeability. Thus, Immunoglobulin (IgG) staining was utilized and evaluated in the hippocampus (Figures 5A, 6A, 7A). BBB permeability, as evaluated through IgG staining, was increased in 6M-MFS mice compared to 6M-CTRL mice in the DG of the hippocampus (Figure 5B), where no differences were seen between 6M-MFS and 12M-CTRL mice. In the CA1 of the hippocampus, IgG staining was not significantly different between experimental groups (Figure 6B). In the CA3 of the hippocampus, IgG staining was increased in 6M-MFS mice compared to 6M-CTRL mice (Figure 7B), where no differences were seen between 6M-MFS and 12M-CTRL mice.

### Microglial morphology is indicative of neuroinflammation due to age and genotype in the hippocampus

**FIGURE 8:**
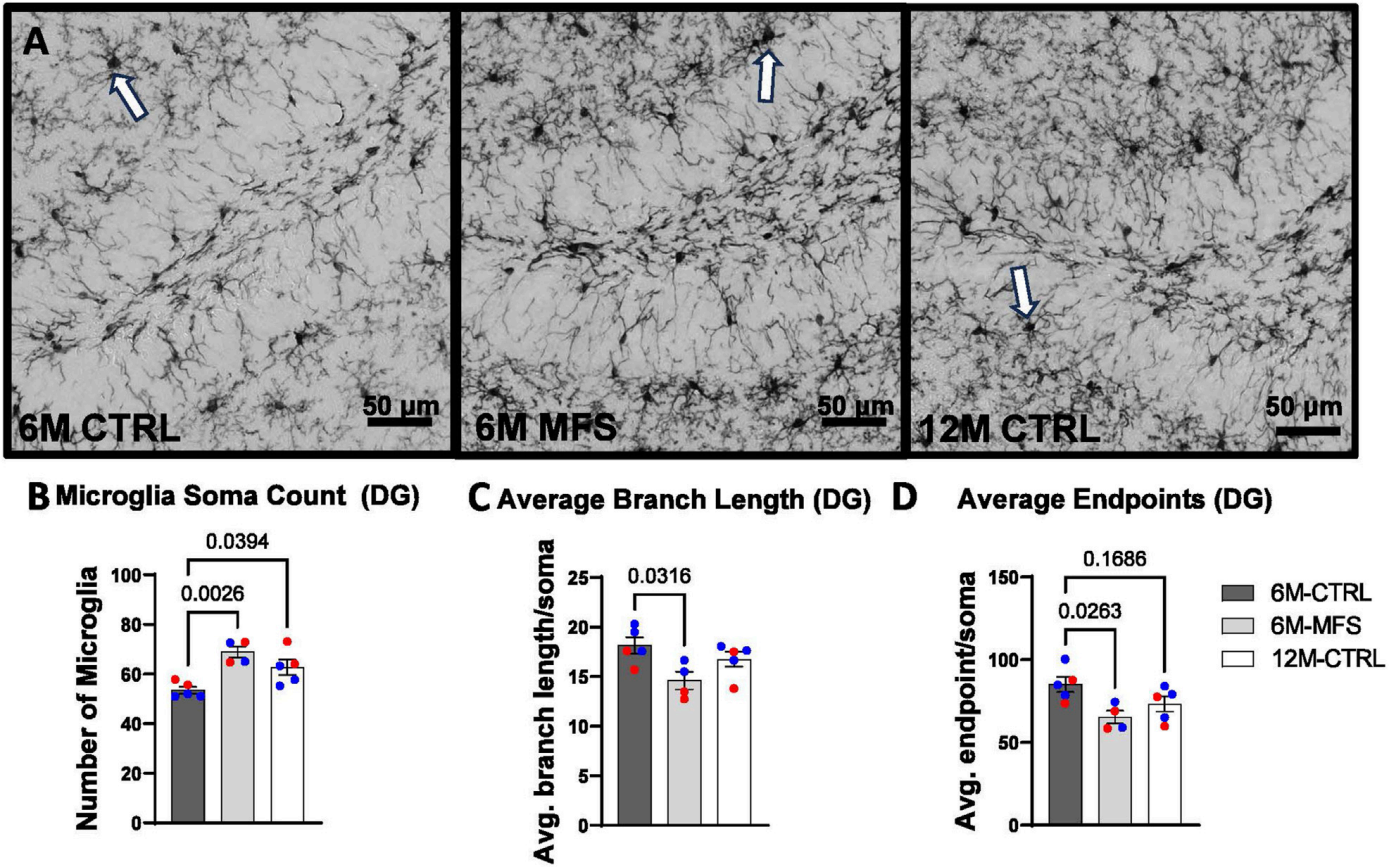
Neuroinflammation is evidenced by increased Iba-1-stained soma count and decreased branch length as a function of genotype and age in the DG of the hippocampus. **(A)** Representative images of Iba-1 staining in the DG of the hippocampus in 6M-CTRL, 6M-MFS, and 12M-CTRL respectively. Examples of positive signals are represented by white arrows. 6M-MFS and 12M-CTRL mice demonstrate increased **(B)** number of microglia, **(C)** 6M-MFS demonstrate decreased branch length, and **(D)** 6M-MFS and 12M-CTRL mice have decreased end point per microglia soma in the DG of the hippocampus, compared to 6M-CTRL mice. Male mice are represented with blue data points and females are represented with red data points.

**FIGURE 9:**
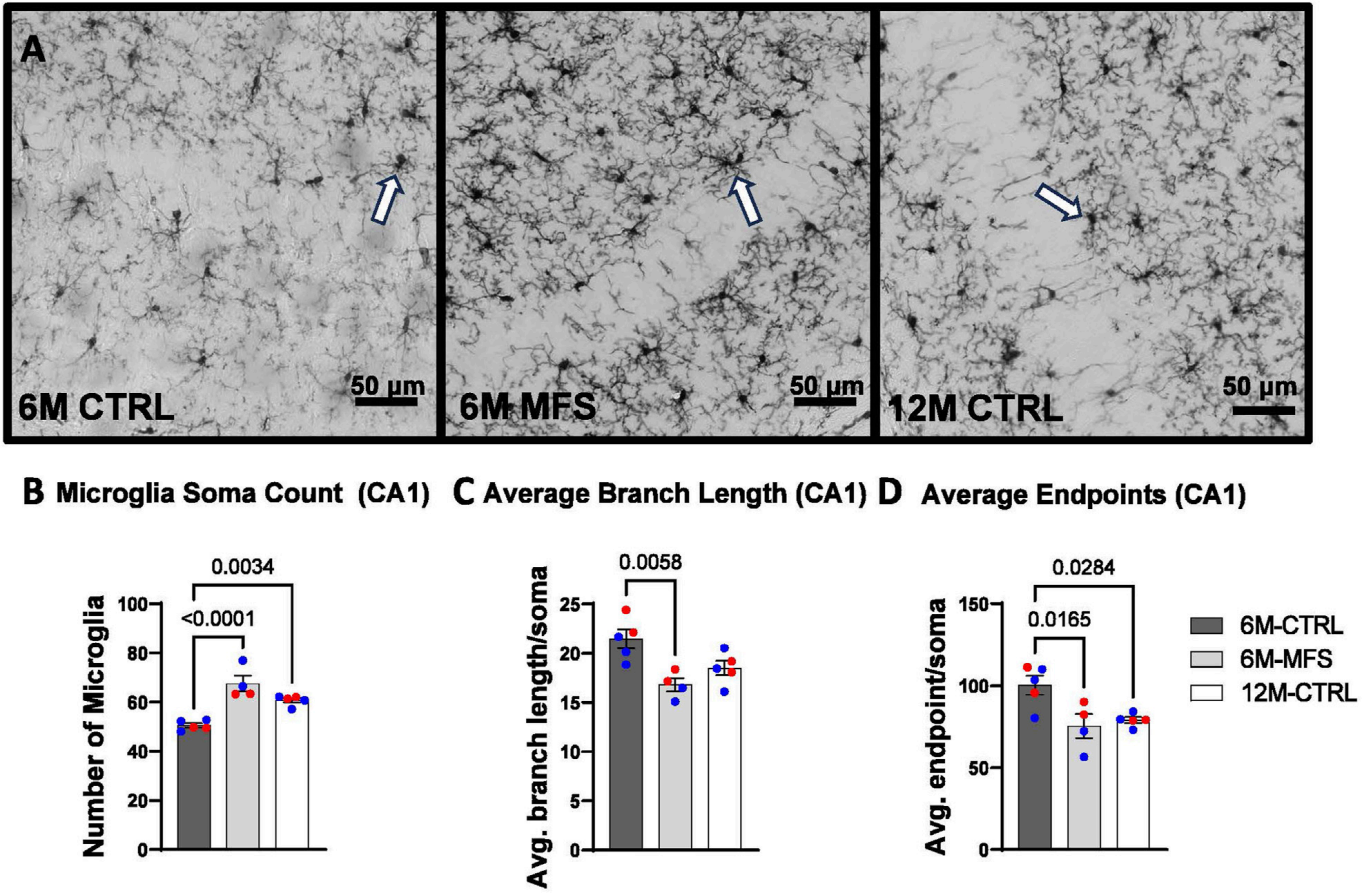
Neuroinflammation is evidenced by increased Iba-1-stained soma count and decreased branch length as a function of genotype and age in the CA1 of the hippocampus. **(A)** Representative images of Iba-1 staining in the CA1 of the hippocampus in 6M-CTRL, 6M-MFS, and 12M-CTRL respectively. Examples of positive signals are represented by white arrows. 6M-MFS and 12M-CTRL mice demonstrate increased **(B)** number of microglia, **(C)** 6M-MFS show decreased branch length, and **(D)** 6M-MFS and 12M-CTRL have decreased end point per microglia soma in the CA1 of the hippocampus, compared to 6M-CTRL mice. Male mice are represented with blue data points and females are represented with red data points.

**FIGURE 10:**
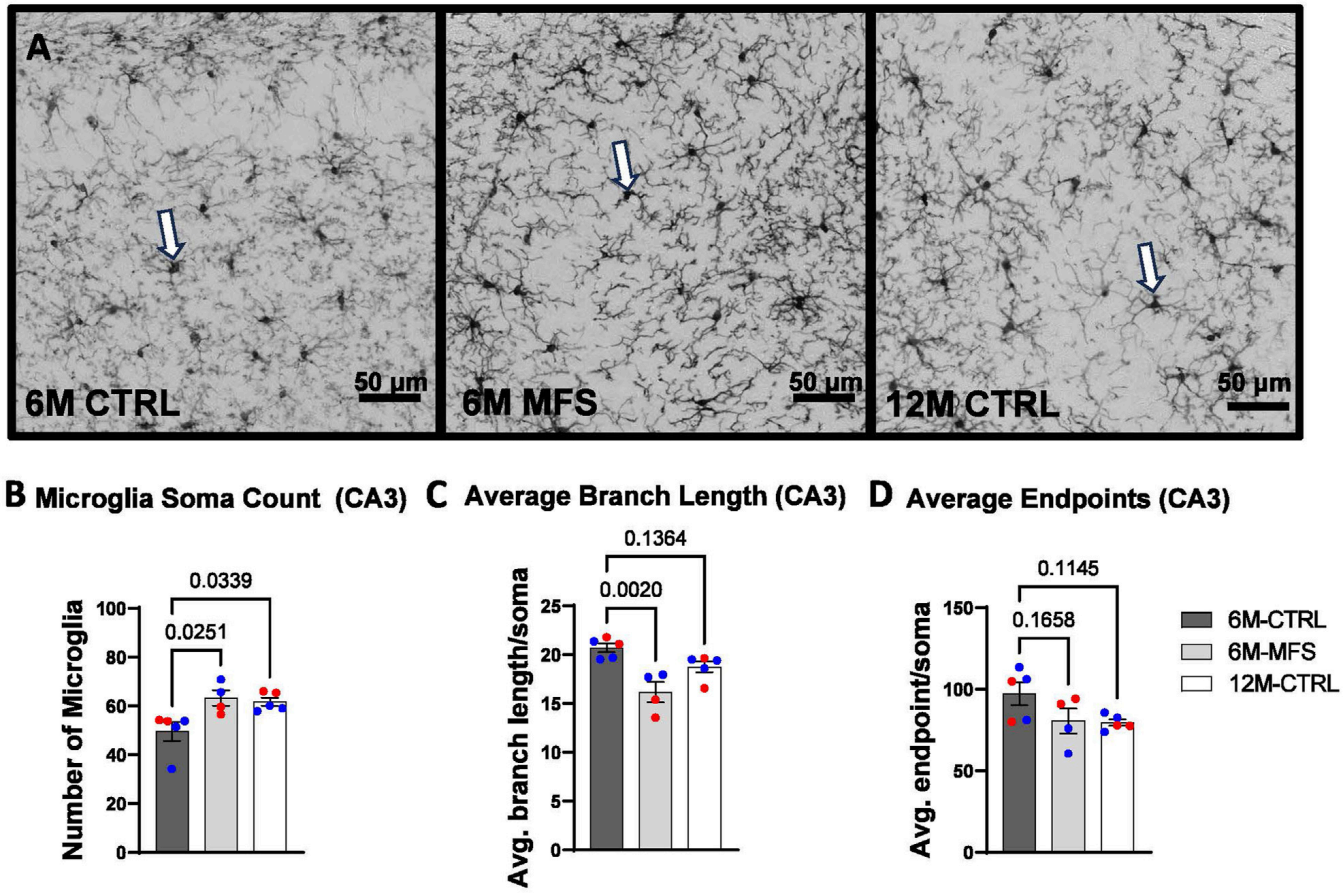
Neuroinflammation is evidenced by increased Iba-1-stained soma count and decreased branch length as a function of genotype and age in the CA3 of the hippocampus. **(A)** Representative images of Iba-1 staining in the CA3 of the hippocampus in 6M-CTRL, 6M-MFS, and 12M-CTRL respectively. Examples of positive signals are represented by white arrows. 6M-MFS and 12M-CTRL mice demonstrate increased **(B)** number of microglia, **(C)** decreased branch length, **(D)** decreased end point per microglia soma in the CA3 of the hippocampus, compared to 6M-CTRL mice. Male mice are represented with blue data points and females are represented with red data points.

To evaluate neuroinflammation, Iba-1 staining, which stains for microglia and infiltrating macrophages, was utilized to visualize microglia soma and morphology (Figures 8A, 9A, 10A). The number of microglia soma in the hippocampus (DG, CA1, and CA3), as well as the morphology of those microglia, were assessed. Previous reports by our laboratory and others have demonstrated that reactive microglia display decreased branch lengths and end points (Beitchman et al., 2020; Bromberg et al., 2020). Skeleton analysis was performed to quantify some of these changes in morphological features in this data set. In the DG of the hippocampus, the number of microglia soma was increased (Figure 8B), while the branch length (Figure 8C) and endpoint (Figure 8D) per soma was decreased when comparing 6M-MFS and 6M-CTRL mice. 12M-CTRL mice were not different than 6M-MFS mice and demonstrated increased number of microglia soma (Figure 8B), no difference in branch length per soma (Figure 8C), and decreased endpoint per soma compared to 6M-CTRL mice in the DG (Figure 8D). In the CA1 of the hippocampus, 6M-MFS demonstrated an increase in the number of microglia soma (Figure 9B), as well as a decrease in the branch lengths (Figure 9C) and endpoints (Figure 9D) per soma, compared to 6M-CTRL mice. Supporting the premature aging phenotype, 12M-CTRL mice demonstrated a similar increase in the number of microglia soma (Figure 9B), yet without a difference in branch length per soma (Figure 9C), as well as decreased endpoints per soma in the CA1, compared to 6M-CTRL mice (Figure 9D). In the CA3 of the hippocampus, 6M-MFS demonstrated increased microglia soma count (Figure 10B), as well as decreased branch length (Figure 10C) and endpoint per microglia (Figure 10D), compared to 6M-CTRL mice. Furthermore, 12M-CTRL similarly demonstrated increased microglial soma count (Figure 10B), decreased branch length (Figure 10C) and endpoints per soma (Figure 10D), compared to 6M-CTRL mice. No differences in iba-1 staining were seen between 6M-MFS and 12M-CTRL throughout the hippocampus.

## Discussion

This report investigated a well-established MFS mouse model with a demonstrated pre-mature cerebrovascular aging phenotype to evaluate corresponding neuropathology. Our findings support that the *Fbn1* mutation contributes to decreased hippocampal microvascular density, increased BBB permeability, and neuroinflammation. Aging similarly contributed to decreased hippocampal microvascular density and neuroinflammation, supporting an MFS-associated premature aging phenotype.

Our lab investigated Glut1, an excepted marker of endothelial cells, as an indirect measure of hippocampal microvasculature in 6M-CTRL, 6M-MFS, and 12M-CTRL mice. The data revealed a decrease in Glut1 staining due to the *Fbn1* mutation as well as normal aging in the DG, CA1, and CA3 areas of the hippocampus. A decrease in cerebral microvascular density, also known as cerebral microvascular rarefaction, has been shown to occur in normal aging, be accelerated in microvascular brain disorders, and play a role in reduction of perfusion (Brown and Thore, 2011; van Dinther et al., 2022). This reduced perfusion can further promote neurological dysfunction and tissue damage (van Dinther et al., 2022). This observed decrease in MFS mice may be correlated with TGF-β′s role in neurovascular development. TGF-β plays a crucial role in the development and differentiation of various cell types within the neurovascular unit (Yamashita et al., 2020). Utilization of a TGF-β inhibitor on human pluripotent stem cells promoted differentiation into endothelial-like cells, where observed blood-vessel-like structures began to form and promoted the synthesis of tight junctions (Yamashita et al., 2020). On the other hand, TGF-β receptor 2 (TβRII) has been demonstrated as essential for proper mediation of cerebral vascular development. Studies involving TβRII knockout mice showed an abnormal cerebral blood vessel phenotype characterized by intracerebral hemorrhage. However, this did not impact the vasculature of other major organs, indicating that TGF-β signaling is crucial for cerebral angiogenesis and development (Nguyen et al., 2011).

Here, we also demonstrated increased BBB permeability in the hippocampus utilizing IgG staining methods due to *Fbn1* mutation. These results are in agreement with previously reported increases in Evans Blue extravasation in the apolipoprotein E (*ApoE*)-deficient mouse model with an *Fbn1* mutation (*ApoE* ^−/−^ *Fbn1* ^C1041G/+^) (Van der Donckt et al., 2015). In the same study, investigators also determined that the *Fbn1* mutation was associated with decreased expression of tight junction proteins claudin-5 and occludin, as well as increased inflammatory cytokines tumor necrosis factor-alpha (TNF-α), MMPs −2/-9, and TGF-β in the choroid plexus (Van der Donckt et al., 2015). Therefore, it is plausible that the increased BBB permeability observed in our study can also be attributed to elevated levels of TGF-β and MMPs and disruptions in tight junctions and structural and functional integrity of BBB. The important function of TGF-β in controlling BBB function is well-established. TGF-β is one of several signaling molecules that cells of the neurovascular unit use to maintain a healthy BBB. Cells of the neurovascular unit both express and secrete TGF-β into the ECM in its latent form, where activation may be initiated by several intracellular events, including but not limited to increased reactive oxygen species (ROS), integrin activation, or protease activity (Alcantara Gomes et al., 2005; Kubiczkova et al., 2012). It has also been documented that TGF-β originating from the periphery can infiltrate the brain and activate its receptors, leading to BBB permeability (Wu et al., 2021). For instance, increased TGF-β signal transduction showed downregulation and destruction of tight junctions through the downstream production of MMP-2, ultimately altering the integrity of the BBB (Wu et al., 2021). While under normal physiological conditions, the activation of MMPs is considered a homeostatic endogenous mechanism in response to damage and injury in the BBB, under chronic conditions, this mechanism can malfunction and contribute to or exacerbate existing damage (Hua et al., 2016).

Increased BBB permeability facilitates the extravasation of ions, proteins, glucose, glutamate, cytokines, and pro-inflammatory molecules into the extracellular space, which can promote neuroinflammation (Candelario-Jalil et al., 2022; Prakash and Carmichael, 2015). In this report, both 6M-MFS and 12M-CTRL mice demonstrated increased Iba-1 positive soma counts in all three regions of the hippocampus and microglial morphology representing reactive microglia. The increased counts were accompanied by observations of cell soma hypertrophy and loss of regional heterogeneity, together, indicative of a neuroinflammatory response. As described above, *ApoE* ^−/−^ *Fbn1* ^C1041G/+^ demonstrated increased TGF-β and MMPs in the choroid plexus, a network of blood vessels in the ventricular space of the brain (Van der Donckt et al., 2015). Furthermore, it has been shown that circulating TGF-β can promote neuroinflammation, where circulating TGF-β originating from the liver during liver failure was found to interact with the brain and increase neuroinflammation and neurological decline (McMillin et al., 2019). Recent studies have confirmed that TGF-β signaling has a biphasic nature, in a manner that in response to an inflammatory stimulus in young mice, TGF-β promotes nitric oxide (NO) secretion in microglia, thus promoting a protective effect; but in aging mice, TGF-β promotes ROS secretion in microglia, thus potentiating neuroinflammation (Tichauer et al., 2014). These studies further demonstrated that the shift from NO induction to ROS induction is due to a change in the TGF-β/SMAD3 pathway, such that over availability of TGF-β actually inhibits the SMAD3 pathway (Tichauer et al., 2014; Tichauer and von Bernhardi, 2012). Therefore, it is plausible that in the mouse model of *Fbn1* mutation, chronic increased TGF- β bioavailability can contribute to the progression of pre-mature aging phenotypes and microglial activation in the brain, specifically in the hippocampus which was evaluated across three distinct regions. In a collaborative study, it was found that activated caspase-3 (ac3) was increased in neurons in the sensory and motor cortical areas of female MFS mice compared to sex and age-matched control mice. Apoptosis was confirmed via TUNEL staining and brain-derived neurotrophic factor levels were increased in both male and female MFS mice. Thus, MFS-associated fibrillin-1 mutation demonstrated an increased susceptibility for neurodegeneration in cortical regions. These findings support further exploration of aging markers, including cellular senescence, as part of future studies. This work is currently under review and is available as a preprint (Esfandiarei et al., 2024). Furthermore, significantly, young MFS mice displayed larger post-ischemic brain injury, evidenced by impaired behaviors, alongside increased neurodegeneration and neuroinflammation. This study uncovers severe brain abnormalities in an MFS mouse model, showing sex- and age-dependent disruptions in brain ECM turnover and redox pathways, along with alterations in cerebrovascular properties. These changes correlate with neuroinflammation and an increased susceptibility to exacerbated post-ischemic damage, stressing the need to monitor neurological risks in MFS patients (Manich et al., 2024).

To our knowledge, this study represents the first report on neuropathology assessment in the mouse model of MFS, potentially paving the way for future analyses in this domain. Throughout this report, we have identified differences in all three regions of the hippocampus, suggesting that future behavioral assays focusing on the evaluation of working memory, spatial navigation, and memory retention are warranted. This direction could provide critical insights into the cognitive impacts of MFS and guide the development of targeted therapeutic strategies.

### Study limitations

The use of the experimental transgenic mouse model provides a systemic approach to manipulate *Fbn1* function and the downstream TGF-β signaling pathway. Changes demonstrated could be due to compensatory mechanisms of chronically upregulated TGF-β. Furthermore, due to multiple isoforms of TGF-β, investigation of these isoforms requires further evaluation. Future studies utilizing a tissue-specific approach to manipulate TGF-β signaling will allow for a better understanding of the role of TGF-β in specific cell types relevant to MFS-associated pre-mature aging of cerebral vasculature and neuropathology.

Furthermore, this study is limited to evaluating a single *Fbn1* mutation associated with MFS, specifically the mutation observed in patients who present with aortic root aneurysm. Consequently, our findings can only be directly applied to this prevalent mutation, rather than being generalizable to all individuals with MFS. This limitation underscores the need for caution when extrapolating our results to the broader MFS patient population.

In this study, we evaluated Iba-1 stained cell morphology and cell counts as indicators of neuroinflammation. While our results suggest a neuroinflammatory response, consistent with findings currently under review, it is important to acknowledge that Iba-1 staining alone does not differentiate between pro-inflammatory and anti-inflammatory microglial activation states (Esfandiarei et al., 2024; Manich et al., 2024). Future studies incorporating cytokine profiling, such as assessing interleukin or tumor necrosis factor levels, or RNA sequencing techniques, would provide a more comprehensive understanding of the neuroinflammatory profiles in MFS mice and their similarity to those in 12-month controls.

It is important to emphasize that although this study incorporated both male and female subjects, it was not designed with the statistical power necessary to assess sex differences across each measured variable. Moreover, since the investigation was not aimed at exploring sex differences, factors that could contribute to variations based on sex as a biological variable, such as hormone levels, were not examined. This approach indicates that while the study acknowledges the inclusion of both sexes, it does not provide insights into how sex-specific biological factors might influence the outcomes or mechanisms under study. Recent preprints and publications support the potential for significant sex differences in peripheral and cerebral outcomes, highlighting the need for further investigation (Jimenez-Altayo et al., 2017; Curry et al., 2024; Esfandiarei et al., 2024; Manich et al., 2024). Last, streamlining the presentation of data by employing a one-way ANOVA to address both research questions provided clarity without compromising the validity of the statistical analysis or the interpretation of the results.

### Conclusion

**FIGURE 11:**
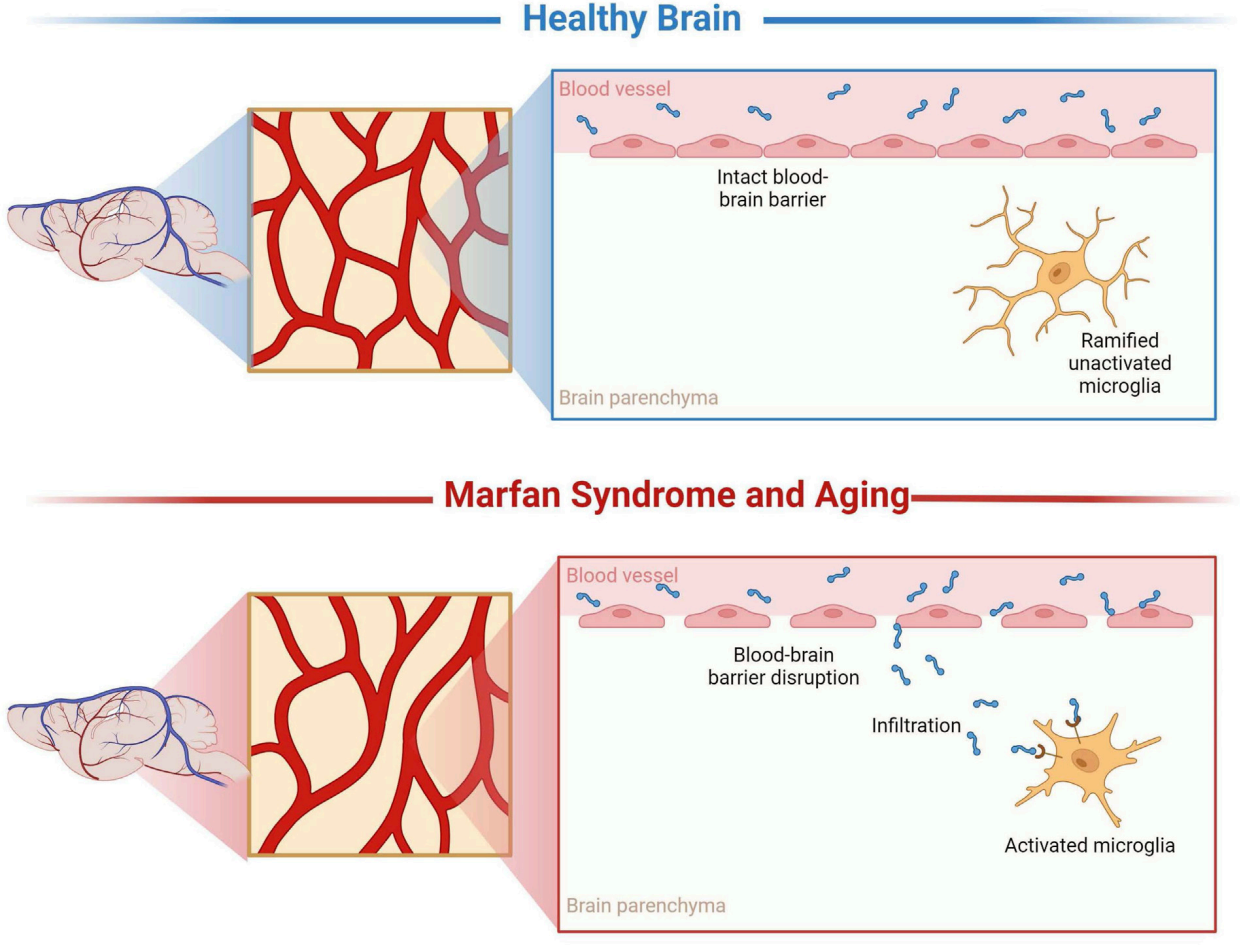
Representation of microvascular density, blood-brain barrier permeability, and neuroinflammatory alterations. These results demonstrate that MFS, similarly, to aging, supports neuropathology through decreased microvascular density, increased BBB permeability, and neuroinflammation compared to healthy controls. Created with BioRender.com.

This study aimed to investigate the unexplored domain of neuropathological changes in connective tissue disorders, specifically MFS. In addition, comparisons were made to middle-aged CTRL mice to elucidate the effects of the premature aging phenotype, characteristic of this connective tissue disorder, on microvascular density, BBB permeability, and neuroinflammation (Figure 11). The findings from this research could lay the groundwork for formulating hypotheses regarding the mechanisms underlying neuropathological alterations observed in a spectrum of disorders that share a similar pathogenesis. This includes conditions characterized by chronically elevated cytokines and MMPs, such as stroke, attention deficit hyperactivity disorder (ADHD), epilepsy, repeated injuries, hypertension, and others, thereby broadening the potential impact of this study beyond MFS to a wider array of neurological conditions.

## Data Availability

The raw data supporting the conclusion of this article will be made available by the authors to qualified researchers upon reasonable request, considering ethical considerations and appropriate use.
